# Interaction between catechol‐O‐methyltransferase polymorphism and childhood trauma in suicidal ideation of patients with post‐traumatic stress disorder

**DOI:** 10.1002/brb3.1733

**Published:** 2020-07-02

**Authors:** Aeran Kwon, Dongil Min, Yourim Kim, Min Jin Jin, Seung‐Hwan Lee

**Affiliations:** ^1^ Clinical Emotion and Cognition Research Laboratory Inje University Goyang Korea; ^2^ Department of Psychiatry Inje University, Ilsan Paik Hospital Goyang Korea

**Keywords:** gene–environment, post‐traumatic stress disorder, suicide

## Abstract

**Introduction:**

Suicidal behavior of post‐traumatic stress disorder (PTSD) patients is influenced by genetic and environmental factors. The catechol‐O‐methyltransferase (*COMT*) gene has been known to be associated with suicidal ideation. The present study aimed to explore the relationship of *COMT* polymorphism, childhood trauma, and suicidal ideation in patients with PTSD.

**Methods:**

Fifty patients with PTSD and 62 healthy controls (HCs) were recruited, and *COMT* variants rs4680 and rs4633 were genotyped through peripheral blood. Psychological assessments such as the childhood trauma questionnaire (CTQ), the scale for suicidal ideation, the clinician‐administered PTSD scale for DSM‐5, and a PTSD checklist were administered. A regression analysis, the Johnson–Neyman technique, and a two‐way analysis of covariance were conducted.

**Results:**

Interaction of *COMT* polymorphism (rs4680, rs4633) and childhood emotional abuse (subscale of CTQ) predicted suicidal ideation in patients with PTSD. Patients with the rs4680 Val/Val genotype, compared to Met carriers genotype, showed higher suicidal ideation when childhood emotional abuse was high. Patients with the rs4633 CC genotype, compared to T carriers genotype, showed higher suicidal ideation when childhood emotional abuse was high.

**Conclusion:**

Our results suggest that vulnerability to suicide could be increased in the Val/Val genotype of *COMT* rs4680 and the CC genotype of rs4633 in patients with PTSD. Moreover, PTSD group with high childhood emotional abuse demonstrated a significantly higher suicidal ideation than did those with low childhood emotional abuse.

## Graphical abstract

Our study demonstrated that the interaction of COMT polymorphism and childhood emotional abuse predicted suicidal ideation in patients with PTSD. Our results suggest that vulnerability to suicide could be increased in the Val/Val genotype of rs4680 and the CC genotype of rs4633 in patients with PTSD.

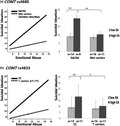

## 1 INTRODUCTION

Exposure to trauma event has been found to be significantly associated with suicide (Stein et al., 2010). An increased risk of suicide and/or suicidal ideation has been repeatedly reported among patients with post‐traumatic stress disorder (PTSD; Bryan & Corso, 2011; Jakupcak et al., 2009; Pompili et al., 2013; Ramsawh et al., 2014). The increased suicidal behavior in PTSD patients remains significant, despite controlling for comorbid psychiatric disorders such as major depressive disorder (Krysinska & Lester, 2010; Wilcox, Storr, & Breslau, 2009).

The genetic component of suicide is an interesting topic and has become increasingly studied. Catechol‐O‐methyltransferase (*COMT*) is one of the candidate genes associated with suicide (Baud et al., 2007; Broekman, Olff, & Boer, 2007; Kia‐Keating, Glatt, & Tsuang, 2007; Nedic et al., 2011) and reported as a risk factor for PTSD (Valente et al., 2011). The *COMT* enzyme is involved in the catalysis and inactivation of catecholamines, such as dopamine. The widely studied rs4680 variant within the *COMT* gene substitutes the amino acid valine (Val) to methionine (Met) at codon 158, which is commonly known as Val158Met polymorphism (Danzi & La Greca, 2018). Both the Met and Val alleles have been found to be associated with PTSD (Kolassa, Kolassa, Ertl, Papassotiropoulos, & De Quervain, 2010; Valente et al., 2011).

Childhood trauma is also strongly associated with suicide attempts later in life (Etain, Henry, Bellivier, Mathieu, & Leboyer, 2008; Lopez‐Castroman et al., 2012, 2015). Childhood trauma may cause the patient with PTSD to experience flashbacks and other PTSD symptoms through helplessness (Wiedemar et al., 2008) or other psychophysiological and personality variables (Orr et al., 2012). Lopez‐Castroman et al. (2012) suggested that PTSD and childhood abuse, particularly when combined, are associated with more severe suicidal behavior and higher suicidal intent. Tang et al. (2018) indicated that psychological maltreatment, such as emotional abuse and emotional neglect, is significantly associated with suicidality even after controlling for PTSD symptom severity. Specifically, emotional abuse influenced the risk of suicidality, directly and indirectly, in PTSD. These results indicate that childhood abuse might be a predictor of future PTSD (Cloitre et al., 2010; Kearney, Wechsler, Kaur, & Lemos‐Miller, 2010; Ozer, Best, Lipsey, & Weiss, 2003).

Although there were a few studies reporting an interaction between the *COMT* Val158Met polymorphism and childhood abuse, they have focused on major depressive disorder, bipolar disorder, anxiety disorders, schizophrenia, and other related disorders (Choi et al., 2018). We could not find studies on suicidal behavior related to the interaction between *COMT* polymorphism and childhood trauma in patients with PTSD. To our knowledge, this is the first study that proposes the relationship between childhood trauma, *COMT* polymorphism, and suicidal ideation in patients with post‐traumatic stress disorder and healthy controls.

We hypothesized that *COMT* polymorphism would interact with childhood trauma to affect suicidal ideation; moreover, we hypothesized that patients with PTSD who had higher scores of childhood abuse would show higher suicidal ideation than those with lower childhood abuse, particularly in those with *COMT* risk allele. It is anticipated that examining childhood trauma and *COMT* polymorphism as risk factors for suicide in patients with PTSD will provide noteworthy implications for its prevention.

## 2 METHODS

### 2.1 Participants

Fifty patients with PTSD (12 males and 38 females) and 62 healthy controls (HCs; 17 males and 45 females) were recruited from the Psychiatry Department of the Inje University Ilsan Paik Hospital in Goyang, Korea. The diagnosis of PTSD was based on the Structured Clinical Interview for Diagnostic and Statistical Manual of Mental Disorders (DSM), Fifth Edition (SCID‐5) by a psychiatrist. Patients were excluded if they had any abnormal brain injury findings by computed tomography or magnetic resonance imaging. HCs were recruited from the local community through flier and posters, were evaluated using the SCID‐5 for psychiatric disorders, and underwent a physical examination by a psychiatrist. HCs were required to have no history of major trauma, such as a serious car accident, combat experience, sexual assault, or serious physical injury, and were not taking medications with potentially psychological disorders. Each participant signed an informed consent form approved by the Institutional Review Board at Inje University Ilsan Paik Hospital before participating (IRB no. 2015‐07‐025).

### 2.2 Psychological measures

#### 2.2.1 Childhood trauma questionnaire

The Korean‐validated version of the childhood trauma questionnaire (CTQ) was used to measure childhood trauma (Yu, Park, Park, Ryu, & Ha, 2009). CTQ consists of five subscales of various childhood traumas, including emotional abuse, physical and sexual abuse, and emotional and physical neglect, as well as another scale for detecting minimization and denial. It is known to be useful in eliciting retrospective reports of childhood maltreatment from young adults (Everson et al., 2008). The CTQ consists of 28 items and is assessed with a 5‐point Likert scale ranging from 1 (“never true”) to 5 (“very often true”). The Korean‐validated version of the CTQ showed adequate reliability and validity (Kim, Park, Yang, & Oh, 2011). The coefficient *α* of the CTQ was .79 in the previous study (Kim et al., 2011) and .91 in the current study. The coefficients of each subtype of the CTQ were .95 (emotional neglect), .93 (physical abuse), .90 (sexual abuse), .92 (emotional abuse), and .65 (physical neglect) in our study.

#### 2.2.2 Scale for suicidal ideation

The Korean‐validated version of the scale for suicidal ideation was used to measure suicidality, which showed adequate reliability and validity (Shin, Park, Oh, & Kim, 1990). The scale consists of 19 items and is assessed with a 3‐point Likert scale ranging from (0–2). The coefficient *α* of this scale was .81 in the previous study (Shin et al., 1990) and .92 in our study.

#### 2.2.3 Clinician‐administered PTSD scale for DSM‐5

The Clinician‐Administered PTSD Scale for DSM‐5 (CAPS) is a structured diagnostic interview that corresponds with the DSM‐5 diagnosis for PTSD, administered by a psychiatrist (Weathers et al., 2018). It consists of 30 items and assesses information about the frequency and severity of PTSD symptoms. This was assessed by standardizing and simplifying the conversion of symptom frequency and intensity ratings into symptom severity ratings (from 0 [“Absent”] to 4 [“Extreme/incapacitating”]) and dichotomous scores (“Yes” or “No”). CAPS showed good internal consistency and validity (Weathers et al., 2018). The coefficient *α* of CAPS severity score was .88 in the previous study and .89 in our study.

#### 2.2.4 Post‐traumatic stress disorder checklist

To examine the severity of PTSD symptoms, the Korean‐validated civilian version of the PTSD checklist (PCL) was administered. The PCL is a self‐report rating scale that measures DSM symptoms of PTSD (Weathers, Litz, Herman, Huska, & Keane, 1993). PCL is often used to diagnose individuals with PTSD or to identify individuals at risk. While CAPS and PCL are highly correlated (Oh et al., 2014; Weathers et al., 2018), discrepancies have also been reported between the overall PTSD symptoms measured by the two questionnaires (Henn‐Haase, 2016). It was indicated that patients with PCL reported more re‐experiencing and fewer symptoms of avoidance of thoughts/feelings, negative emotions, detachment, and restricted affect compared to CAPS. The Korean‐validated version of the PCL showed adequate reliability and validity (Oh et al., 2014). It consists of 17 items and assessed with a 5‐point Likert scale ranging from 1 (“not at all”) to 5 (“extremely”). The coefficient *α* was .93 in the previous study and .97 in our study.

#### 2.2.5 Hospital anxiety and depression scale

The Korean‐validated version of the hospital anxiety and depression scale (HADS) was used to evaluate anxiety and depression symptoms. It consists of seven items for describing anxiety and seven items for determining depression (Oh, Min, & Park, 1999), and it is assessed with a 4‐point Likert scale, ranging from 0 (no problems) to 3 (maximum distress). The Korean‐validated version of HADS showed adequate reliability and validity (Oh et al., 1999). The coefficients of each subtype of HADS were .89 (anxiety) and .86 (depression) in the previous study and .93 (anxiety) and .86 (depression) in the current study.

### 2.3 *COMT* genotyping

All participants had their blood sampled to extract DNA using a NanoDrop^®^ ND‐1000 UV‐Vis Spectrophotometer. Genomic DNA was then diluted to a 5 ng/μl concentration in 96‐well polymerase chain reaction (PCR) plates. TaqMan™ Single Nucleotide Polymorphism (SNP) Genotyping Assays (Thermo Fisher Scientific) were obtained, and the probes were labeled with FAM or VIC dye at the 5′ end and a minor‐groove binder and nonfluorescent quencher at the 3′ end. PCR was done in 5 μl of a mixture containing 2 μl of a DNA sample, 0.125 μl of each TaqMan™ SNP Genotyping Assay (Thermo Fisher Scientific), 2.5 μl of TaqMan™ Genotyping Master Mix (Thermo Fisher Scientific), and 0.375 μl of distilled water. Amplification and detection were done with a detection system (QuantStudio 12K Flex Real‐Time PCR System; Thermo Fisher Scientific) with the profile of 50°C for 2 min and 95°C for 10 min, followed by 60 cycles of 95°C for 15 s and 6°C for 1 min. After the PCR amplification, allelic discrimination was performed using the same machines (QuantStudio 12K Flex Real‐Time PCR System), which was considered an endpoint plate read. The QuantStudio 12K Flex Software calculated the fluorescence measurements made during the plate read and plotted Rn values based on the signals from each well. Finally, the analyzed plates were used to perform automatic or manual allele calls.

There were three positive and one negative control samples for each plate, and we confirmed positive controls with a clustering image. Our intragenomic DNA (gDNA) samples of known genotypes were used for positive control. We calculated genotype frequencies for each individual polymorphism and evaluated the Hardy–Weinberg equilibrium to check the data quality and genotype error. The chi‐squared test was used to compare the observed numbers of each genotype with those expected for the population following chi‐square distribution with one degree of freedom (Weir & Cockerham, 1996). All statistical tests and visualization of differentially expressed genes were conducted using R version 3.3.3.

Twenty‐three patients with PTSD were found to have Val/Val (GG) genotype, and 27 participants with PTSD were Met carriers (GA+AA) in *COMT* rs4680. In HCs with *COMT* rs4680, 34 participants were of Val/Val genotype, and 28 participants were Met carriers. Twenty‐four patients with PTSD were of CC genotype, and 26 participants with PTSD displayed T carriers (CT+TT) in *COMT* rs4633. In HCs with *COMT* rs4633, 33 participants were of CC genotype, and 29 participants were CT+TT.

### 2.4 Statistical analyses

Normality was tested using the skewness and kurtosis. Skewness <2.0 and kurtosis <7.0 were considered to be moderately normally distributed (Curran, West, & Finch, 1996). All variables in our results were within the range of normal distribution. After checking for normality, a regression analysis using SPSS Macro PROCESS for SPSS (version 2.16.3; Hayes, 2013) was performed to examine the moderating effect of childhood trauma on the path between *COMT* polymorphisms and suicidality. Years of education was controlled as a covariate. In contrast, sex and age were not included as covariates, since there were no differences of sex and age between PTSD and HCs (Table 1). Depression and anxiety scales were not controlled since negative mood is considered as a main symptom of PTSD (Dai et al., 2017; Flory & Yehuda, 2015).

**TABLE 1:** Demographics, clinical characteristics, and *COMT* variants of participants

	PTSD (*N* = 50)	HCs (*N* = 62)	*t* or *χ* ^2^	*p‐*value
	*M* (*SD*) or *N* (%)			
Demographics				
Age (years)	41.84 (12.57)	43.82 (14.37)	0.767	.445
Sex (M/F)	12 (24)/38 (76)	17 (27.40)/45 (72.60)	0.169	.681
Education (years)	12.16(3.53)	13.71 (3.40)	2.358	.020
Clinical characteristics				
PCL	46.38(16.08)	10.90 (9.21)	−14.569	<.001
CAPS—severity	38.18(10.15)	6.73 (7.94)	−18.404	<.001
CAPS—no. of symptoms	12.98(3.29)	2.19 (2.99)	−18.173	<.001
HADS—anxiety	12.62(4.48)	4.65 (2.11)	−11.600	<.001
HADS—depression	11.90 (3.86)	4.90 (2.44)	−11.143	<.001
CTQ	50.12 (22.21)	39.85 (13.33)	−2.877	.005
Emotional neglect	12.20 (6.93)	9.90 (4.78)	−1.992	.050
Physical abuse	10.66 (7.02)	8.18 (4.28)	−2.193	.031
Sexual abuse	7.54 (4.60)	6.27 (2.78)	−1.711	.091
Emotional abuse	10.50 (6.70)	7.10 (3.50)	−3.250	.002
Physical neglect	9.22 (4.28)	8.40 (3.39)	−1.127	.262
*COMT* genotypes frequency				
rs4680				
Val/Val (GG)	23 (46.00)	34 (54.84)	1.049	.592
Val/Met (GA)	25 (50.00)	25 (40.32)		
Met/Met (AA)	2 (4.00)	3 (4.84)		
rs4633				
CC	24 (48.00)	33 (53.23)	0.420	.811
CT	24 (48.00)	26 (41.93)		
TT	2 (4.00)	3 (4.84)		

Abbreviations: CAPS, clinician‐administered PTSD scale for DSM‐5; C*OMT*, catechol‐O‐methyltransferase; CTQ, childhood trauma questionnaire; HADS, hospital anxiety and depression scale; HCs, healthy controls; PCL, PTSD checklist for DSM‐5; PTSD, post‐traumatic stress disorder.

Subsequently, the Johnson–Neyman technique (Hayes, 2013) was applied to investigate the regions in which the moderating variable had a significant effect. The Johnson–Neyman technique aligns the moderating variable in a continuous manner and computes the regions of significance for interactions by examining the significance between the predictor and outcome variables (Preacher, Curran, & Bauer, 2006).

Finally, a two‐way analysis of covariance (ANCOVA) was used to probe the direction of the interactions. The moderating variable was divided into high and low groups at the point in which the moderating effect was first observed. Years of education was controlled for as a covariate. All significant levels were set at *p* < .05 (two‐tailed), and all statistical analyses were performed using SPSS version 21 (IBM Inc.).

## 3 RESULTS

### 3.1 Descriptive statistics

Demographics, clinical characteristics, and *COMT* variants in PTSD and HCs groups are presented in Table 1. Years of education of patients with PTSD were significantly lower than HCs (12.16 ± 3.53 vs. 13.71 ± 3.40, *p* = .020), and scores of PCL, CAPS severity, and some subscales on the CTQ (physical abuse and emotional abuse) were significantly higher in the PTSD group than HCs. There was no significant difference in genotype frequency of *COMT* (rs4680 and rs4633) between the groups. Also, there was no significant interaction between *COMT* genotype and sex/age.

### 3.2 Moderating effect

The results of the hierarchical regression analysis are shown in Table 2. For *COMT* rs4680 polymorphism, rs4680 did not predict suicidal ideation in model 1 (*β* = −0.233, *t* = −1.621, *p* < .112). In model 2, there were significant main effects of rs4680 and childhood emotional abuse (*β* = −0.298, *t* = −2.185, *p* = .034; *β* = 0.376, *t* = 2.818, *p* = .007, respectively). In model 3, there was a significant interaction between rs4680 and childhood emotional abuse (*β* = −1.193, *t* = −2.095, *p* = .042).

**TABLE 2:** Regression analysis examining moderating effects of childhood Emotional Abuse (EA) on the path between *COMT* polymorphism (rs4680, rs4633) and suicidal ideation

Predicting variables	PTSD (*N* = 50)						HCs (*N* = 62)					
	*R* ^2^	Δ*R* ^2^	Δ*F*	*β*	*t*	*p*	*R* ^2^	Δ*R* ^2^	Δ*F*	*β*	*t*	*p*
1	.067	.067	1.680			.197	.026.	.026	0.780			.463
Education				−0.073	−0.510	.613				−0.080	−0.595	.554
rs4680				−0.233	−0.1.621	.112				−0.116	−0.861	.393
2	.204	.137	7.939			.007	.262	.236	18.522			.000
Education				−0.052	−0.390	.698				−0.040	−0.340	.735
rs4680				−0.298	−2.185	.034				−0.042	−0.352	.735
EA				0.376	2.818	.007				0.495	4.304	.000
3	.275	.071	4.389			.042	.292	.030	2.430			.125
Education				−0.043	−0.335	.739				−0.049	−0.420	.676
rs4680				0.295	0.576	.567				0.335	1.244	.219
EA				1.383	2.779	**.008**				0.995	2.922	**.005**
rs4680 × EA				−1.193	−2.095	**.042**				−0.597	−0.1.559	.125
1	.078	.078	1.981			.149	.015	.015	0.438			.648
Education				−0.080	−0.566	.574				−0.109	−0.825	.413
rs4633				−0.255	−1.794	.079				−0.034	−0.254	.800
2	.220	.142	8.359			.006	.260	.246	19.254			.000
Education				−0.062	−0.467	.643				−0.056	−0.479	.364
rs4633				−0.322	−2.404	.020				0.015	0.127	.900
EA				0.383	2.891	.006				0.502	4.388	.000
3	.288	.068	4.291			.044	.261	.000	0.024			.878
Education				−0.051	−0.401	.690				−0.061	−0.499	.620
rs4633				0.112	0.455	.651				0.053	0.193	.848
EA				1.370	2.776	**.008**				0.555	1.530	.132
rs4633 × EA				−1.173	−2.071	**.044**				−0.064	−0.154	.878

Abbreviations: *COMT*, catechol‐O‐methyltransferase; HCs, healthy controls; PTSD, post‐traumatic stress disorder.

Bold value indicates a statistically significant correlation with a *p*‐value less than 0.05.

For *COMT* rs4633 polymorphism, in model 1, rs4633 did not predict suicidal ideation (*β* = −0.255, *t* = −1.794, *p* < .079). In model 2, there were significant main effects of rs4633 and childhood emotional abuse (*β* = −0.322, *t* = −2.404, *p* = .020; *β* = 0.383, *t* = 2.891, *p* = .006, respectively). In model 3, the interaction between rs4633 and childhood emotional abuse was significant (*β* = −1.173, *t* = −2.071, *p* = .044).

However, there were no significant interactions in other variables in patients with PTSD. Additionally, there were no significant interaction effects in HCs.

### 3.3 Probing an interaction

The Johnson–Neyman analysis was executed. In *COMT* rs4680 (Table 3), the conditional effect was significant when the childhood emotional abuse score was above 9.233. It implies that the interaction between childhood emotional abuse and the rs4680 on suicidal ideation was significant only when the childhood emotional abuse score exceeded 9.233.

**TABLE 3:** Johnson–Neyman analysis for the moderating effects of childhood emotional abuse (EA) on the path between *COMT* polymorphism and suicidal ideation in patients with PTSD (*N* = 50)

*COMT* rs4680 → suicidal ideation							*COMT* rs4633 → suicidal ideation						
EA	Effect	*SE*	*t*	*p*	LLCI	ULCI	EA	Effect	*SE*	*t*	*p*	LLCI	ULCI
5.000	−1.258	2.679	−0.469	.641	−6.654	4.139	5.000	−1.685	2.646	−0.637	.528	−7.014	3.644
9.000	−4.087	2.121	−1.927	.060	−8.359	0.186	8.000	−3.766	2.161	−1.742	.088	−8.119	0.587
9.233	−4.252	2.111	−2.014	.050	−8.504	0.000	8.679	−4.237	2.104	−2.014	.050	−8.474	0.000
10.000	−4.794	2.098	−2.285	.027	−9.019	−0.569	9.000	−4.460	2.084	−2.140	.038	−8.658	−0.262
25.000	−15.404	5.501	−2.800	.008	−26.485	−4.324	25.000	−15.560	5.442	−2.859	.006	−26.521	−4.599

Note

LLCI, ULCI: lower and upper limits within the 95% confidence interval of the moderating effects.

In *COMT* rs4633 (Table 3), the conditional effect was significant when the childhood emotional abuse score was above 8.679. It implies that the interaction between childhood emotional abuse and the rs4633 on suicidal ideation was significant only when the childhood emotional abuse score exceeded 8.679.

The two‐way ANCOVA was executed for probing the direction of the interaction between *COMT* polymorphism and childhood emotional abuse. In *COMT* rs4680, the genotype (Val/Val vs. Met carriers) was set and the childhood emotional abuse score was divided into high and low groups based on the score of 9.233. Subsequently, patients with PTSD were divided into four groups. In *COMT* rs4633, the genotype (CC vs. T carriers) was set and the childhood emotional abuse score was divided into high and low groups based on the score of 8.679. Subsequently, patients with PTSD were divided into four groups.

**FIGURE 1:**
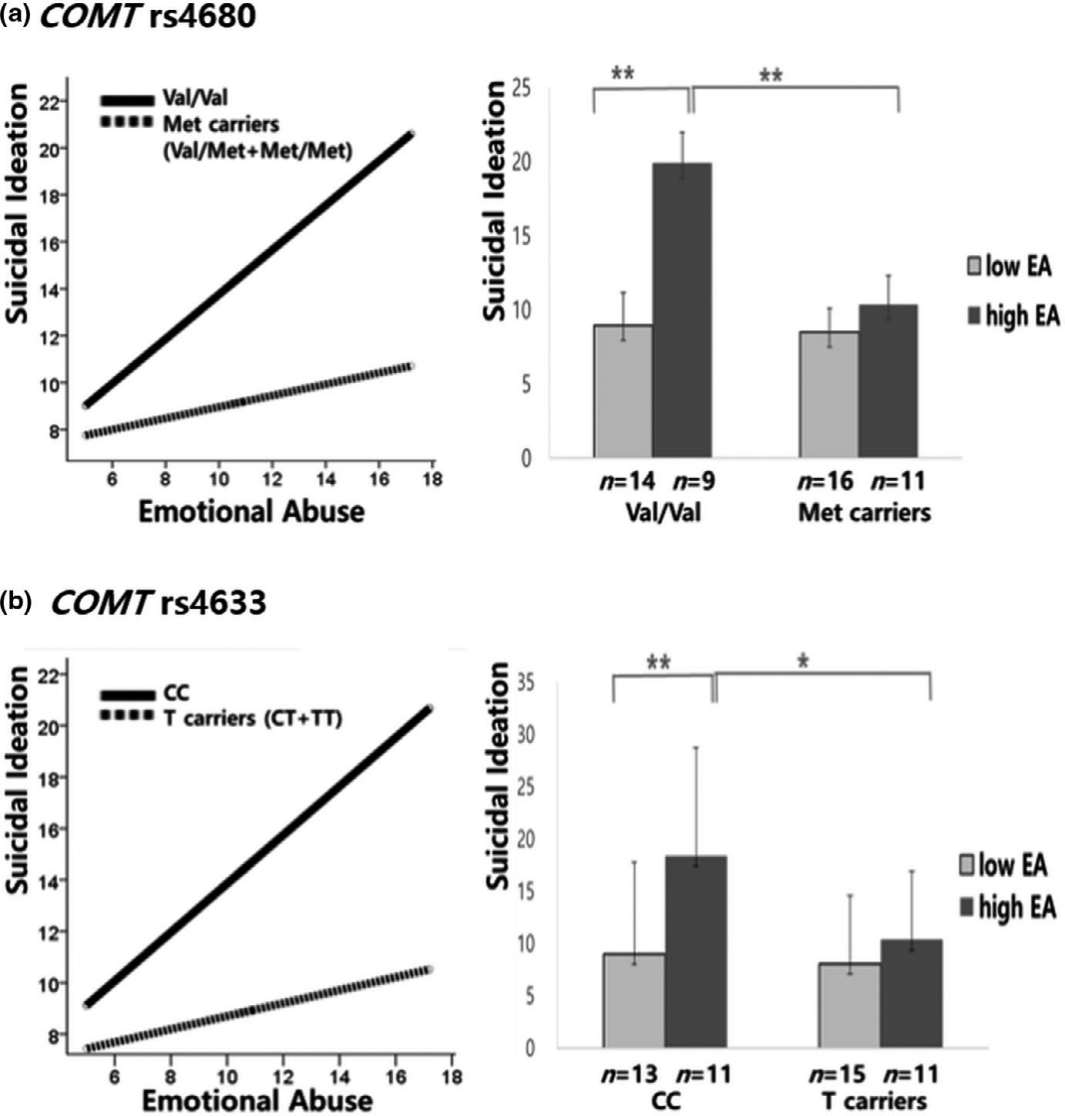
Interaction effects between the childhood emotional abuse and *COMT* polymorphism (rs4680, rs4633) on suicidal ideation. *p < .05; **p < .01

For PTSD patients with Val/Val genotype of rs4680, the results indicated that suicidal ideation was significantly higher in the high childhood emotional abuse group than the low childhood emotional abuse group (*F* = 11.237, *p* = .003). However, in Met carriers, suicidal ideation was not significantly different between high and low childhood emotional abuse groups (*F* = 0.537, *p* = .471; Figure 1a). For PTSD patients with CC genotype of rs4633, suicidal ideation was significantly higher in the high childhood emotional abuse group than in low childhood emotional abuse group (*F* = 8.398, *p* = .008). However, in T carriers, suicidal ideation was not significantly different between high and low childhood emotional abuse groups (*F* = 0.796, *p* = .381; Figure 1b).

## 4 DISCUSSION

The present study aimed to explore the relationship between childhood trauma, *COMT* polymorphism (rs4680 and rs4633), and suicidal ideation in patients with PTSD and HCs. Our results showed that the interaction of *COMT* polymorphism and childhood emotional abuse predicted suicidal ideation in patients with PTSD. Patients with the Val/Val genotype of rs4680 and the CC genotype of rs4633 showed a higher suicidal ideation when the childhood emotional abuse was high. Our results suggest that vulnerability to suicide could be increased in the Val/Val genotype of rs4680 and the CC genotype of rs4633 in patients with PTSD.

Our results demonstrated that interaction between *COMT* polymorphism and childhood emotional abuse predicted suicidal ideation in patients with PTSD. Exposure to childhood trauma has been known to be an important risk factor for suicidal behavior (Brodsky & Stanley, 2008; Dube et al., 2001; Miller, Esposito‐Smythers, Weismoore, & Renshaw, 2013). In addition, exposure to early‐life trauma has shown to strongly increase the susceptibility to develop PTSD as adults (Yehuda et al., 2010). However, a genetic predisposition which is modulated or triggered by environmental factors could be a significant influencing factor on suicidal behavior (Mandelli & Serretti, 2013; Saveanu & Nemeroff, 2012). *COMT* polymorphism is one of the most common genes that have been studied in mental illness. Choi et al. (2018) found that rs4680 in the *COMT* gene was associated with suicide attempts in patients with mood disorders. Bernegger et al. (2018) found an association between *COMT* polymorphism and suicidal behavior in major depressive disorder and bipolar disorder patients who experienced childhood maltreatment. *COMT* rs4680, as well as interactive effects between this gene and environmental adversities, has been associated with anxiety and panic symptom (Asselmann et al., 2018; Baumann et al., 2013). In sum, our findings support the previous notion that *COMT* gene–environment interactions could be related to suicidal ideation in various mental illnesses.

In our study, participants with the *COMT* rs4680 Val/Val genotype, compared to Met carriers genotype, showed a higher suicidal ideation when childhood emotional abuse was high. Previous studies also reported that the rs4680 Val allele is related with higher risk for psychopathologies and biological abnormalities (Baekken, Skorpen, Stordal, Zwart, & Hagen, 2008; Caspi et al., 2005; Craddock, Owen, & O'Donovan, 2006; Olsson et al., 2007; Thapar et al., 2005). Schulz‐Heik et al. (2011) indicated that the rs4680 Val/Val genotype and the associated decrease in intrasynaptic dopamine might increase the vulnerability to dystrophic effects of trauma on the human brain. The Val/Val genotype was associated with high levels of dissociation (Savitz et al., 2008) and anger (Perroud et al., 2010) in individuals exposed to higher levels of childhood trauma. Our result is consistent with prior findings in the sense that the people with rs4680 Val/Val genotype might be more vulnerable for psychopathologies in early childhood trauma experiences.

In Korean studies, the Val/Val genotype and Val carriers were more frequent in male suicide attempters (among patients with major depressive disorder, schizophrenia, and bipolar disorder) than in male HCs (Lee & Kim, 2011). Choi et al. (2018) indicated the association between the *COMT* Met/Met genotype and suicide attempts in patients with mood disorders (117 with depressive disorder and 99 with bipolar disorder). This observed inconsistency might be caused by the different mental illnesses which were recruited in each Korean study. Our study focused on suicidality in patients with PTSD, but other studies have focused on patients with various mental illnesses (Choi et al., 2018; Lee & Kim, 2011). Our study considered the interaction of *COMT* rs4680 and childhood emotional abuse, but previous studies investigated only the main effect of independent variables on each *COMT* polymorphism such as childhood trauma, current alcohol consumption, and related depression scores (Choi et al., 2018; Lee & Kim, 2011).

Another possible interpretation is based on the mouse model (Scheggia et al., 2018), which demonstrates that mice with relatively increased *COMT* activity (i.e., mice with Val genotype) display enhanced aversive remote memories. It was known that *COMT* genetic variants affected prefrontal activation during long‐term memory encoding and retrieval (Bertolino et al., 2006; Schott et al., 2006; Wimber et al., 2011). This study might indicate a possible role of *COMT* in psychiatric conditions associated with inappropriate retention of past aversive memories, such as PTSD. It suggests that individuals carrying *COMT* Val genotype could have higher vulnerability and more severe symptoms of PTSD.

In our study, the CC genotype of rs4633, compared to T carriers, showed a higher suicidal ideation when the childhood emotional abuse was high in patients with PTSD. A recent study reported that rs4633 was related to total PTSD symptom score based on DSM‐5 (Goenjian et al., 2015). In particular, the symptom “negative changes in thoughts and mood” was regarded to be relevant. Our result is in line with this study in the sense that suicidal ideation is one of the severe negative thoughts and mood in PTSD symptoms. Although our results indicate the possibility that the CC genotype of rs4633 increases the vulnerability of suicidal ideation in patients with PTSD, there is a need for additional studies to enhance the understanding of this genotype effect.

Conversely, our results fail to demonstrate the interaction between *COMT* polymorphism and childhood emotional abuse on suicidal ideation in the HCs group. In the HCs group, childhood emotional abuse appeared to predict suicidal ideation, but the interaction effect with *COMT* polymorphism was not significant. This might be due to the difference in the ratio of *COMT* genotype between patients with PTSD (Val/Val; 40.4%) and HCs (Val/Val; 59.6%). In addition, these differences could be derived from the different levels of severity of childhood trauma between the two groups. The HCs group, compared to PTSD, did not have severe childhood traumatic experiences or recent trauma to cause PTSD symptoms.

Additionally, there was neither a significant interaction between *COMT* genotype and sex, nor a significant group difference in sex between PTSD and HCs in current study. Previous studies reported sex‐dependent effects related to COMT genetic variants (Papaleo, Sannino, Piras, & Spalletta, 2015; Sannino et al., 2014, 2017). Such discrepancy may be due to the different methods used and the ethnicity‐specific genetic difference.

There are some limitations in this study. First, the CTQ and its subscales may not precisely reflect the participants' traumatic childhood experiences because it is a subjective and retrospective self‐report. Second, suicidal ideation and suicide attempts were considered as one continuous process in our study, which is important to re‐examine as suicidality could be a heterogeneous construct (Stein et al., 2010).

In conclusion, our study demonstrated that the interaction of *COMT* polymorphisms (rs4680, rs4633) and childhood emotional abuse predicted suicidal ideation in patients with PTSD. Our results suggested that vulnerability to suicide could be increased in the Val/Val genotype of *COMT* rs4680 and the CC genotype of rs4633 in patients with PTSD. Moreover, PTSD group with high childhood emotional abuse demonstrated a significantly higher suicidal ideation than did those with low childhood emotional abuse.

## CONFLICT OF INTEREST

The authors declare no conflicts of interest.

## AUTHOR CONTRIBUTIONS

A.K. conducted all statistical analyses and wrote the manuscript. D.M. and Y.K. collected the data. M.J.J. edited the manuscript. S.H.L. designed the study, supervised its analysis, and edited the manuscript. All authors discussed the results and implications and commented on the manuscript at all stages.

### Peer Review

The peer review history for this article is available at https://doi.org/10.1002/brb3.1733.

## Data Availability

The data that support the findings of this study are available from the corresponding author, S.H.L., upon reasonable request.
